# Cross-contamination in canine and feline dietetic limited-antigen wet diets

**DOI:** 10.1186/s12917-018-1571-4

**Published:** 2018-09-12

**Authors:** Elena Pagani, Maria de los Dolores Soto del Rio, Alessandra Dalmasso, Maria Teresa Bottero, Achille Schiavone, Liviana Prola

**Affiliations:** Department of Veterinary Sciences, University of Turin, L.go Braccini, 2, 10095 Turin, Grugliasco (TO) Italy

**Keywords:** Adverse food reaction, Cross contamination, Pet food, Polymerase chain reaction, Food safety

## Abstract

**Background:**

Adverse food reactions (AFRs) are defined as abnormal responses to an ingested food or food additive. Diagnosis and treatment of AFRs consist of the complete elimination of these ingredients in the dietary trial. Previous studies have demonstrated the presence of undeclared ingredients in commercial limited-antigen dry food diets that can compromise the results and efficacy of dietary elimination trails. The aim of this study was to assess a selection of commercial canine and feline dietetic limited-antigen wet foods for the potential cross-contamination of animal proteins from origins not mentioned on the label.

**Results:**

Eleven canine and feline dietetic limited-antigen wet foods (9 novel animal protein foods, 1 vegetarian and 1 hydrolyzed) were analyzed by polymerase chain reaction (PCR) to detect the presence DNA of animal and vegetal origins. PCR analysis confirmed the contamination of 6 of the 11 (54.5%) limited-antigen wet diets with undeclared animal protein. One of these 6 diets was solely composed of animal protein sources completely unrelated to those declared on the label. None of the foods containing horse meat or fish were contaminated, and neither were the vegetarian or the hydrolyzed food products. Moreover, the results show that had zoological class primers only been used to check for cross-class contaminations, as are generally used in the pet food industry for in-house checks, the apparent contamination rate would have been significantly underestimated: less than 20% (3/11), instead of the actual rate of 54.7% using species-specific primers.

**Conclusion:**

This study reveals a high rate of cross-contamination in dietetic limited-antigen wet canine and feline foods, as previously described for dietetic dry limited-antigen foods (reported to be more than 80%). These results add new fuel to the discussion about the potential causes underlying the failure of elimination diets, since animal protein contaminants may actually be present in the commercial dietetic limited-antigen diets. AFRs may therefore occur as a result of inadequate practices in the pet food industry.

## Background

Adverse food reactions (AFRs) are defined as abnormal responses to an ingested food or food additive [1, 2]. When an AFR is suspected, its diagnosis should always be based on a complete and accurate dietary history, clinical signs and, in particular, on the results of a dietary elimination test, i.e., an “elimination and re-introduction diet” [3]. Indeed the gold standard method to diagnose an AFR consists of feeding the animal a limited-antigen diet until the abatement of clinical signs (elimination diet) and then reintroducing the diet previously fed to demonstrate the recurrence of symptoms (provocation diet) [3]. Other tests, such as skin testing, skin patch testing, serologic tests measuring food allergen-specific serum IgE, and gastric biopsy tests have already been shown to have low sensitivity and specificity [3–5]. The elimination diet should contain protein and carbohydrate sources that the animal has never eaten before the trial and should be administered for a period of at least 8–10 weeks [6]. Clinical signs of gastrointestinal disease usually improve within 2 weeks, whereas cutaneous clinical signs may take up to 8 to 12 weeks to respond to dietary change. For the elimination diet, veterinarians can use either commercial veterinary prescription diets, such as limited-antigen diets or hydrolyzed diets, or home-cooked diets [6]. Commercially available limited-antigen diets typically include proteins from venison, quail, rabbit and duck and are generally combined with alternative carbohydrate sources, such as green peas, rice and potatoes. A hydrolyzed diet can provide an alternative to the novel protein diet; they contain small peptides of molecular weights below 3 kDa, which are easily digestible and associated with low antigenic stimulation [7], but their efficacy has not always been confirmed [6, 8–12]. In the case of no evident improvement in clinical signs during a dietary trial, AFR cannot be excluded, because residual allergenic fragments may remain in the hydrolyzed diet and contamination may have occurred during the production process of limited-antigen diets [13]. In each of these cases, the correct diagnosis of AFR would be compromised. Many studies have already demonstrated the presence of undeclared ingredients in limited-antigen dry diets, dry and wet physiological foods, vegetarian and vegan diets, supplements and treats for pets, such as microscopic bone fragments, proteins, detected by enzyme-linked immunosorbent assay (ELISA), and deoxyribonucleic acid (DNA), detected by polymerase chain reaction (PCR), reverse transcription PCR, multiplex PCR or PCR-restriction fragment length polymorphism [14–24]. Considering that the production technologies involved in dry and wet pet food manufacturing are distinct, the aim of the present study was to investigate whether the high rate of contamination found in commercial limited-antigen dry diets [13] is also applicable to the antigen-limited wet diets often used in dietary elimination trials for the diagnosis and therapy of AFR in pets.

## Methods

### Samples

Eleven canine and feline antigen-limited wet diets (10 novel protein diets and 1 hydrolyzed diet) produced by 5 different pet food manufacturers (2 Italian and 3 international) were obtained from veterinary clinics. All of the products used in this study were declared as dietetic complete pet food, specifically intended for particular animal nutritional purpose of reduction of ingredients and nutrient intolerances, according to the European law 2008/38/EC [25]. For each sample, the product’s label was carefully studied to identify all protein sources of animal origin and of vegetal origin in the case that the product was declared to be vegetarian. The product’s brand, description, therapeutic indications, animal species, feed material declaration, additives lists, analytical constituents, instruction for proper use, net weight, lot number, expiry date, and the name and address of the business operator responsible for its labelling were recorded.

Each pet food product was randomly assigned a unique three-digit sample identification number. Samples were then submitted to the polymerase chain reaction (PCR) to identify DNA belonging to 3 zoological classes (mammalian, avian and fish). Based on these results, PCR analyses for specific animal species (quail, rabbit, duck, horse, deer, turkey, chicken, domestic ruminant, tuna and swine) were performed according to the zoological classes testing positive. The limited-antigen diet composed of vegetal protein only was also tested using PCR to identify vegetal protein origin.

### DNA extraction

To extract DNA from pet food samples, the DNeasy Blood and Tissue Kit (Qiagen, Hilden, Germany) was used following the manufacturer’s instructions. Negative controls (water) were included in each extraction. DNA concentration was determined by spectrophotometry (Nanodrop ND1000; NanoDrop Technologies Inc., Wilmington, DE, USA).

### Species identification protocol

Previously published PCR primers (Table 1) designed to recognise species-specific regions of mitochondrial DNA were used to test for the presence of quail, rabbit, duck, horse, turkey, chicken, ruminant and swine products. Deer and tuna were identified using minisequencinq protocols (Table 1)**.** For horse, a specific real time PCR assay was run in a 7300 Real-Time PCR System (Applied Biosystems, Foster City, CA, USA). The PCR amplifications were performed in an ABI 2720 thermocycler (Applied Biosystems, Foster City, CA,USA). Minisequencing was performed on an ABI 310 Genetic Analyzer (Applied Biosystems, Foster City, CA, USA). All amplification protocols were performed following the published protocols (Table 1).

**Table 1:** Primers used for the detection of species specific and zoological class DNA

Primer	PCR product (bp)	References
Detection of species-specific DNA		
Chicken	95	Martín et al., 2007 [32]
Deer	232	La Neve et al., 2008 [33]
Duck	64	Martín et al., 2007 [32]
Horse	147	Kesmen et al., 2009 [34]
Quail	129	Rojas et al., 2010 [35]
Rabbit	160	Walker et al., 2004 [36]
Domestic Ruminant	104	Dalmasso et al., 2004 [15]
Swine	108	Meyer et al., 1995 [37]
Tuna	132	Bottero et al., 2007 [38]
Turkey	122	Martín et al., 2007 [32]
Detection of zoological class DNA		
MAMMALIAN	117	Chiappini et al., 2005 [39]
FISH	224	Dalmasso et al., 2004 [15]
POULTRY	183	Dalmasso et al., 2004 [15]
VEGETABLE	132	Little DP, 2014 [39]

## Results

Eleven canine and feline antigen-limited wet diets were collected and submitted to PCR to test for the presence of DNA from 3 different zoological classes (mammalian, avian and fish), and subsequently for DNA from specific animal species as well as for DNA of vegetal origin. Table 2 shows the animal protein sources as listed on the product labels and the results obtained by species-specific PCR. Discrepancies between the results expected and those obtained from PCR were observed in 6 out of the 11 sampled diets. Of the 6 showing discrepancies, 5 were contaminated with protein from additional animal species and 1 was wholly composed of animal protein sources completely unrelated to those declared on the label. Of these 5 contaminated with additional animal species, 2 contained DNA belonging to 2 different zoological classes, i.e., inter-zoological class contaminations: Sample n.6 declared rabbit as its unique animal protein source on the label, but PCR detected DNA belonging to 2 different zoological classes: 2 avian (turkey and chicken) and 1 mammalian (rabbit). Sample n.11 declared duck as its unique animal protein source on the label, but PCR again detected DNA belonging to 2 different zoological classes: 3 avian (duck, chicken, and turkey) and 1 mammalian (pork).. In the remaining 3 samples contaminated with additional animal species, the contaminating species belonged to the same zoological class. Sample n.1 declared turkey as its unique animal protein source on the label, whereas PCR analysis detected DNA from turkey and chicken. Sample n.3 declared deer as its unique animal protein source on the label, but PCR analysis detected DNA from another domestic ruminant in addition to deer. Sample n.8 declared quail as its unique animal protein source on the label, but PCR analysis confirmed the presence of another avian species (not covered by the species-specific PCR primers listed in Table 1) in addition to quail. Finally, the label for sample n.7 indicated duck as its unique animal protein source, but PCR analysis was only able to detect DNA from turkey, chicken and horse, (and thus a case of inter zoological class contamination); no traces of duck DNA were found. In the remaining 5 sample diets, PCR analysis confirmed the animal protein species sources declared on the label.

**Table 2:** List of declared animal protein sources and PCR results expressed as zoological class and species in 11 antigen-limited wet diets

Samples	Declared animal protein source- Zoological class	Declared animal protein source-species	Declared fat source	PCR analysis results- Zoological class	PCR analysis results-Species
1	A	Turkey	Vegetal	A	Turkey Chicken
2	M	Horse	Chicken	M	Horse
3	M	Deer	Fish	M	Deer Domestic Ruminant
4	None	None	Vegetal	None	Vegetal^a^
5	F	Tuna	Fish	F	Tuna
6	M	Rabbit	Vegetal	M A	Rabbit Chicken Turkey
7	A	Duck	Vegetal	A M	Turkey Chicken Horse
8	A	Quail	Vegetal	A	Quail Another Avian
9	M	Horse	Vegetal	M	Horse
10	A	Hydrolyzed Chicken	Fish and Vegetal	A	Hydrolyzed Chicken
11	A	Duck	Fish and Vegetal	A M	Duck Turkey Chicken Swine

*A* avian, *F* fish, *M* mammalian

^a^ Diet declared as “vegetarian”, with no animal species declared, but positive results for a vegetal primer

## Discussion

The elimination diet is currently the most important and reliable diagnostic test for evaluating and diagnosing AFRs in dogs and cats [26]. The test comprises two phases: 1) the accurate selection by veterinarians of a specific limited-antigen diet (commercial or homemade, based on a novel or hydrolyzed protein), avoiding ingredients previously fed; 2) the meticulously administration of this selected diet by the pet’s owner. Limited-antigen diets should have the following characteristics: eliminate the potential allergens from the diet, be easy to use, readily available and reasonably priced [8]. Commercial limited-antigen diets are most frequently used due to their convenience and relative low cost, but unfortunately a recent study reports that limited-antigen dry diets should not be considered reliable due to their high degree of contamination with other sources of animal protein not declared on the label [14]. The authors found more than 80% of such diets to be contaminated [14]. The present study used PCR analysis to assess for the potential cross-contamination of antigen-limited wet diets with animal proteins not mentioned on the label and confirmed a high rate of contamination (54.5%) despite the different technologies involved in wet animal food production and the different feed materials commonly used. Discrepancies between the labelling declarations and the results obtained by PCR analyses were observed in 6 out of the 11 diets sampled; in particular, of these 6 contaminated diets, 5 were contaminated with additional animal species and 1 was composed of animal protein sources completely unrelated to that declared on the label. These results provide an important input to the debate on the potential causes behind why elimination diets may fail; i.e., if animal protein contaminants are widespread in the commercial dietetic limited-antigen pet foods, inadequate practices in the pet food industry could be implicated as a cause of AFRs. The pet food industry has a legal obligation to produce safe food for its consumers (Article 1, Regulation No. 767/2009) [27]. Based on the Directive 2008/38/EC, dietetic limited-antigen diets are indicated for the reduction of ingredient and nutrient intolerances in dogs and cats and for that purpose they should be formulated with selected protein sources and/or carbohydrate sources; in the current EU legislation, the tolerance of analytical traces of other animal proteins is not discussed [25]. Regarding allergens, the pet food industry has not been obliged to embrace allergen risk management, as the human food industry has had to. Food allergen risk should be considered in parallel with physical, chemical and microbiological risks. For example, in the manufacturing of baby foods intended for human consumption, production lines are often dedicated to the manufacture of a single product composed of specific antigenic ingredients. Furthermore, the labelling of human foodstuffs is obliged to inform the consumer of the possible presence of potential allergens, and allergen management has become an integral part of the Hazard Analysis and Critical Control Points (HACCP). With regard to human food, the EU has implemented regulations that require the presence of certain potential allergens, which are present as ingredients, to be declared by manufacturers (EU Directive 2007/68/EC and EU Regulation 1169/2011, which came into force in 2014) [28, 29]. Thus, the management of animal protein traces in the pet food industry providing dietetic limited-antigen foods should be seen as an integral part of the management of existing food safety procedures and should take into account all the operations, from feed materials to manufacturing and the packaging of the final products. The adoption of good practices in pet food manufacturing will allow veterinarians to make more informed choices in relation to elimination trials, which is essential for the correct diagnosis and treatment of AFRs in dogs and cats.

Indeed, the high prevalence of AFRs in dogs and cats is an important reason why allergen management should become an integral part of the food safety management system in the pet food industry. In canine populations, AFRs were diagnosed in 1.69% of dogs presented to a veterinary teaching hospital in a single year [30]. In cats, the prevalence of AFRs was described to be less than 1%, but higher in cats with skin diseases, for whom it ranged from 3 to 6% [31]. Thus the high incidence of animal protein cross-contamination in dietetic pet foods, highlighted by our results and by many previous studies [14–24], indicates a highly unsatisfactory situation. Animal protein cross-contamination could occur at two levels in the pet food industry: i) during the production of the feed materials (animal by-products); or ii) during the actual production of pet food (limited-antigen diets).

The possibility of intentional cross-contamination of either feed materials or final products is unlikely considering the similar or sometimes higher cost of the undeclared animal species identified in our samples, such as the horse protein found in sample n.7 which declared duck as its unique animal protein source. Intentional cross-contamination might occur in situations of limited availability of some selected protein sources, such as duck, for which availability cannot always be guaranteed for pet food production with high meat inclusion rates.

In order to ensure a high level of feed safety and to improve transparency, provisions should be added as an integral aspect of good practice. Although every effort could be taken by the pet food industry to eliminate the risk posed by the unintended presence of food allergens, in many businesses it is virtually impossible to produce a zero risk product. In many manufacturing premises, production lines dedicated to the manufacture of a single product are not always feasible or practical for economic or logistical reasons. Therefore, the cleaning of shared equipment, processing lines and the local environment becomes a key element in allergen control. Manufacturers are presently required to define the cleaning procedures and cleaning schedules appropriate for their facilities. But they are not required to ensure or to demonstrate that the cleaning programme is effective and performing properly through the use of specific and accurate analyses, such as PCR-food analysis. Multiple lines are more frequently used for the production of commercial dry pet food than for the production of wet food. This could justify the lower contamination rate of our wet samples (54.5%) compared with that for dry foods analysed by other authors, described as being over 80% [14]. Our methodology was also more sensitive, involving the use of species-specific primers rather than zoological class primers only, as used by previous authors [14]. Had we used zoological class primers only, the apparent rate of contamination would have been greatly underestimated: lower than 20% instead of 54.5% as obtained using species-specific primers. Indeed, had we used zoological class primers, sample 7 would not have resulted as being a totally misleading diet, and samples 1, 3, 8 and 11 would not have resulted as being contaminated at all.

One of the most interesting results obtained in our study using the species-specific PCR was that diets with protein sources derived from horse and fish (tuna) were always clean (100% as declared, with no contaminations). These data might imply that the different feed material production techniques affect the potential for cross-contamination by other animal protein sources; moreover, they highlight and shift the focus from the pet food manufacturers to the suppliers of feed materials (animal by-products). It seems that when separate lines are required for the slaughter, transformation and distribution of animal products (as is the case for horse meat and fish), the final by-products are also cleaner. In the past, some authors also focused their attention on lipid sources of animal origin. In the present study, the animal lipid sources declared on the label were never found to be species-specific or zoological class PCR positive [14]. It would be interesting if processing standards similar to those used for hydrolyzed protein production (Section 5 D Reg. 142/2011) were also applied in manufacturing plants processing animal proteins intended for limited-antigen diets [28]. Adherence to these specific processing requirements could help avoid cross-contaminations between different animal protein sources. Good practices that would help avoid cross-contaminations could include: the use of dedicated containers and means of transport maintained in a clean state (cleaned, washed and/or disinfected after each use) for the carriage of animal protein sources of a single animal species; the clear and total separation of the manufacturing plant areas involved in the processing of animal proteins intended for limited-antigens diets, from reception until dispatch; the correct setting up and management of the equipment used for product processing, including separate processing lines and cleaning procedures to exclude the risk of cross-contamination.

The results of this study are very important for pet food producers of limited-antigen diets. They indicate the need for improvements to be made in the selection of feed material suppliers and the type of checks installed throughout the production chain before final products are introduced onto the market. Importantly, our results indicate that the type of checks used should involve PCR analyses for species-specific classes and not just zoological class.

## Conclusion

In conclusion, the high incidence of contaminations found in the antigen-limited wet diets analyzed in our study confirm the severity of the problem as previously highlighted for dietetic limited-antigen dry diets [14]. These results provide a new slant on the debate regarding the causation of elimination diet failure as they suggest that the practices in place in the pet food industry lead to the presence of animal protein contaminants in commercial dietetic limited-antigen diets, which could, in turn, result in AFRs. In order to ensure food safety standards of these commercial diets and guarantee the efficacy of the diagnosis and treatment of AFRs in dogs and cats, general best practices should be adopted by the pet food industry. The management of cross contaminant animal proteins in limited-antigen diets may be addressed at three levels: (1) The feed materials and supply chain: establish an appropriate policy for assessing the allergen status of the feed materials, such as the identification of species-specific allergens by PCR analysis in feed materials received from suppliers before their unloading in the factory; (2) manufacturing equipment and processes: integrate cross-contamination risk assessment management into production processes at different levels; this should involve cleaning programs for staff, equipment, production and packaging lines and a method for validating efficacy that involves PCR analyses and clearly identification of the re-work programs in order to be tracked; (3) labelling: in the interests of transparency, information should be added to the label regarding the possibility of cross contamination antigens. This additional information should be mandatory for dietetic antigen-limited foods and include the analytical limit of detection and testing method.

## Abbreviations

A: Avian
AFR: Adverse food reactions
DNA: Deoxyribonucleic acid
F: Fish
FA: Food allergy
M: Mammalian
PCR: Polymerase chain reaction

## References

[1] Anderson JA (1985). Food allergy and food intolerance. ASDC J Dent Child.

[2] Guilford WG, Strombeck DR, Guilford WG (1996). Adverse reactions to food. Small Animal Gastroenterology.

[3] Scott DW, Millet WH, Griffin CE (2001). Mueller & Kirk’s Small Animal Dermatology.

[4] Ishida R, Masuda K, Kurata K, Ohno K, Tsujimoto H (2004). Lymphocyte blastogenic responses to inciting food allergens in dogs with food hypersensitivity. J Vet Intern Med.

[5] Picco F, Zini E, Nett C, Naegeli C, Bigler B, Rüfenacht S, Meng E (2008). A prospective study on canine atopic dermatitis and food-induced allergic dermatitis in Switzerland. Ve dermatol.

[6] Gaschen FP, Merchant SR (2011). Adverse food reactions in dogs and cats. Vet Clin North Am Small Anim Pract.

[7] Cave NJ (2006). Hydrolyzed protein diets for dogs and cats. Vet Clin North Am Small Anim Pract.

[8] Loeffler A, Soares-Magalhaes R, Bond R, Lloyd DH (2006). A retrospective analysis of case series using home-prepared and chicken hydrolysate diets in the diagnosis of adverse food reactions in 181 pruritic dogs. Vet Dermatol.

[9] Biourge VC, Fontaine J, Vroom MW (2004). Diagnosis of adverse reactions to food in dogs: efficacy of a soy-isolate hydrolyzate-based diet. J Nutr.

[10] Loeffler A, Lloyd DH, Bond R, Kim JY, Pfeiffer DU (2004). Dietary trials with a commercial chicken hydrolysate diet in 63 pruritic dogs. Vet Rec.

[11] Roitel O, Bonnard L, Stella A, Schiltz O, Maurice D, Douchin G, Jacquenet S, Favrot C, Bihain BE, Couturier N.Detection of IgE-reactive proteins in hydrolysed dog foods. Vet Dermatol. 2017;28(6):589–e143. 10.1111/vde.12473 28770578

[12] Olivry T, Bizikova P (2010). A systematic review of the evidence of reduced allergenicity and clinical benefit of food hydrolysates in dogs with cutaneous adverse food reactions. Vet Dermatol.

[13] Ricci R, Hammerberg B, Paps J, Contiero B, Jackson H (2010). A comparison of the clinical manifestations of feeding whole and hydrolysed chicken to dogs with hypersensitivity to the native protein. Vet Dermatol.

[14] Ricci R, Granato A, Vascellari M (2013). Identification of undeclared sources of animal origin in canine dry foods used in dietary elimination trials. J Anim Physiol Anim Nutr (Berl)..

[15] Dalmasso A, Fontanella E, Piatti P, Civera T, Rosati S, Bottero MT (2004). A multiplex PCR assay for the identification of animal species in feedstuff. Mol Cell Probes.

[16] Myers MJ, Farrell DE, Heller DN, Yancy HF (2004). Development of a polymerase chain reaction-based method to identify species-specific components in dog food. Am J Vet Res.

[17] Wang HC, Lee SH, Chang TJ, Wong ML (2004). Examination of meat components in commercial dog and cat feed by using polymerase chain reaction-restriction fragment length polymorphisms (PCR-RFLPs) technique. J Vet Med Sci.

[18] Raditic DM, Remillard RL, Tater KC (2011). ELISA testing for common food antigens in four dry dog foods used in dietary elimination trials. J Anim Physiol Anim Nutr.

[19] Parr JM, Remillard RL (2014). Common confounders of dietary elimination trials contain the antigens soy, pork, and beef. J Am Anim Hosp Assoc.

[20] Pegels N, Gonzalez I, Garcia T, Martin R (2014). Avian-specific real-time PCR assay for authenticity control in farm animal feeds and pet foods. Food Chem.

[21] Hsieh MK, Shih PY, Wei CF, Vickroy TW, Chou CC (2016). Detection of undeclared animal by-products in commercial canine canned foods: comparative analyses by ELISA and PCRRFLP coupled with slab gel electrophoresis or capillary gel electrophoresis. J Scie Food Agric.

[22] Maine IR, Atterbury R, Chang KC (2015). Investigation into the animal species contents of popular wet pet foods. ACTA Vet Scan.

[23] Okuma TA, Hellberg RS (2015). Identification of meat species in pet foods using a real-time polymerase chain reaction(PCR) assay. Food Control.

[24] Kanakubo K, Fascetti AJ, Larsen JA (2017). Determination of mammalian deoxyribonucleic acid (DNA) in commercial vegetarian and vegan diets for dogs and cats. J Anim Physiol Anim Nutr (Berl).

[25] European Commission (2008). Commision directive of 3 March 2008 establishing a list of intended uses of animal feedingstuffs for particular nutritional purposes. (2008/38/EC). Official J Eur Communities.

[26] Pali-Schöll I, De Lucia M, Jackson H, Janda J, Mueller RS, Jensen-Jarolim E (2017). Comparing immediate-type food allergy in humans and companion animals-revealing unmet needs. Allergy.

[27] European Parliament and Council (2009). Regulation (EC) No 767/2009 of 13 July 2009 on the placing on the market and use of feed, amending European Parliament and Council Regulation (EC) No 1831/2003 and repealing Council Directive 79/373/EEC, Commission Directive 80/511/EEC, Council Directives 82/471/EEC, 83/228/EEC, 93/74/EEC, 93/113/EC and 96/25/EC and Commission Decision 2004/217/EC. (767/2009/CE). Official J Eur Communities.

[28] European Commission (2011). Commision directive of 25 Febraury 2011 implementing regulation (EC) no 1069/2009 of the European Parliament and of the council laying down health rules as regards animal by-products and derived products not intended for human consumption and implementing council directive 97/78/EC as regards certain samples and items exempt from veterinary checks at the border under that directive (142/2011/CE). Official J Eur Communities.

[29] European Commission (2007). Commision directive of 27 november 2007 amending annex IIIa to directive 200/13/EC of European Parliament and of the council as regards certain food ingredients. (2007/68/CE). Official J Eur Communities.

[30] Proverbio D, Perego R, Spada E, Ferro E (2010). Prevalence of adverse food reactions in 130 dogs in Italy with dermatological signs: a retrospective study. J Small Anim Pract.

[31] Olivry T, Mueller RS (2017). Critically appraised topic on adverse food reactions of companion animals (3): prevalence of cutaneous adverse food reactions in dogs and cats. BMC Vet Res.

[32] Martín I, García T, Fajardo V, López-Calleja I, Hernández PE, González I, Martín R (2007). Species-specific PCR for the identification of ruminant species in feedstuffs. Meat Sci.

[33] La Neve F, Civera T, Mucci N, Bottero MT (2008). Authentication of meat from game and domestic species by SNaPshot minisequencing analysis. Meat Sci.

[34] Kesmen Z, Gulluce A, Sahin F, Yetim H (2009). Identification of meat species by Taqman-based real –time PCR assay. Meat Sci.

[35] Rojas M, González I, Pavon MA, Pegels N, Lago A, Hernández PE, Garía T, Martín R (2010). Novel TaqMan real-time polymerase chain reaction assay for verifying the authenticity of meat and commercial meat products from game birds. Food Add Contam.

[36] Walker JA, Hughes DA, Hedges DJ, Anders BA, Laborde ME, Shewale J, Sinha SK, Batzer MA (2004). Quantitative PCR for DNA identification based on genome-specific interspersed repetitive elements. Genomics.

[37] Meyer R, Höfelein C, Lüthy J, Candrian U (1995). Polymerase chain reaction-restriction fragment length polymorphism analysis: a simple method for species identification in food. J AOAC Int.

[38] Bottero MT, Dalmasso A, Cappelletti M, Secchi C, Civera T (2007). Differentiation of five tuna species by a multiplex primer-extension assay. J Biotechnol.

[39] Chiappini B, Brambilla G, Agrimi U, Vaccari G, Aarts HJ, Berben G, Giambra V (2005). Real-time polymerase chain reaction approach for quantitation of ruminant-specific DNA to indicate a correlation between DNA amount and meat and bone meal heat treatments. J AOAC Internat.

